# Simulated coal spill causes mortality and growth inhibition in tropical marine organisms

**DOI:** 10.1038/srep25894

**Published:** 2016-05-13

**Authors:** Kathryn L. E. Berry, Mia O. Hoogenboom, Florita Flores, Andrew P. Negri

**Affiliations:** 1 College of Science and Engineering, James Cook University, Townsville, Queensland, Australia; 2 Australian Institute of Marine Science, Townsville, Queensland, Australia; 3 AIMS@JCU, Australian Institute of Marine Science and James Cook University, Townsville, Queensland, Australia; 4 ARC Centre of Excellence for Coral Reef Studies, James Cook University, Townsville, Queensland, Australia

## Abstract

Coal is a principal fossil fuel driving economic and social development, and increases in global coal shipments have paralleled expansion of the industry. To identify the potential harm associated with chronic marine coal contamination, three taxa abundant in tropical marine ecosystems (the coral *Acropora tenuis*, the reef fish *Acanthochromis polyacanthus* and the seagrass *Halodule uninervis*) were exposed to five concentrations (0–275 mg coal l^−1^) of suspended coal dust (<63 μm) over 28 d. Results demonstrate that chronic coal exposure can cause considerable lethal effects on corals, and reductions in seagrass and fish growth rates. Coral survivorship and seagrass growth rates were inversely related to increasing coal concentrations (≥38 mg coal l^−1^) and effects increased between 14 and 28 d, whereas fish growth rates were similarly depressed at all coal concentrations tested. This investigation provides novel insights into direct coal impacts on key tropical taxa for application in the assessment of risks posed by increasing coal shipments in globally threatened marine ecosystems.

The international trade of coal is highly dependent on transportation by sea, and coal shipments continue to increase on a global scale^1^. Recent scientific, political and public opinion has raised concerns regarding the increase in coal mining and shipping adjacent to sensitive tropical coastal environments, including World Heritage listed sites such as the Great Barrier Reef (GBR)^2^. To date, major concerns about these activities have pertained to increased dredging to facilitate port access for coal vessels, and the burning of coal increasing greenhouse gas emissions. However, growth in seaborne coal trade has also been accompanied by increased shipping accidents that have potential to cause widespread damage to marine ecosystems. For instance, the groundings of the bulk coal carriers *Castillo de Salas* (Spain, 1986), *Eurobulker IV* (Italy, 2000) and *MV Smart* (South Africa, 2013) released between 17,000 and 100,000 tons of unburnt coal into the marine environment^3^,^4^. Calm weather conditions helped prevent 68,000 tons of coal on board the grounded *Shen Neng I* from spilling onto the GBR in 2010^5^.

Despite the occurrence of these large-scale incidents over a period of several decades, there is currently no scientific consensus on the levels at which unburnt coal becomes a threat to the health of tropical marine organisms. Although seaborne coal trade is highest in tropical regions (Indonesia and Australia)^1^, studies of coal impacts on marine environments have generally been conducted in temperate regions where, for example, long-term colliery waste contamination was linked with declines in species richness and diversity^6^. The paucity of knowledge on coal impacts in tropical environments means that we are unable to competently assess the potential threats of accidental release of coal into tropical marine environments.

Other forms of particulate matter contamination in seawater (e.g., sediment) can reduce the growth and survival of tropical marine organisms by reducing light penetration into water and by smothering tissues through direct deposition of particles onto organisms^7^,^8^. Similarly, direct pathways for organism harm by coal are likely to include suspended particles, increased light attenuation from turbidity, and smothering of sessile benthic organisms, leading to reduced photosynthesis and feeding^9^. In addition, coal may contain contaminants such as polycyclic aromatic hydrocarbons (PAHs) and trace metals, and a fraction of these contaminants can be released from coal dust into the surrounding seawater^9^,^10^,^11^. Metals can be toxic to marine species by disrupting enzyme activity and membrane structure, but the effects of metals are highly dependent on speciation and bioavailability^12^. PAHs affect organisms via non-specific narcosis^13^ and can be carcinogenic and mutagenic to marine life^14^. Sub-lethal chronic effects may include reduced growth, decreased fecundity and reproductive failure; however, the response to PAHs also varies greatly with bioavailability and the capacity of organisms to detoxify during metabolism^15^.

We evaluated lethal and sub-lethal coal concentrations by quantifying the effects of suspended coal dust and coal dust deposition on key demographic rates (growth, mortality) of coral (*A. tenuis*), fish (*A. polyacanthus,* spiny chromis) and seagrass (*H. uninervis*). The potential effects of coal on key representative species from the tropics, measured here for the first time, is critical for the development of appropriate risk assessments and policy development associated with the safe and sustainable shipment of coal through the GBR and other tropical ecosystems of high ecological value.

## Results and Discussion

### Water Quality in Experimental Treatments

Experimental treatments of suspended and settling coal particles mimicked five broad pulse intensities (ranging from 0–275 mg coal l^−1^, Table 1) lasting 28 d. Attenuation of light in coal treatments ranged from 44–99%, relative to control values (Table 1). Coal deposition rates ranged from 11–241 mg cm^−2 ^d^−1^ in sediment traps and 2–46 mg cm^−2 ^d^−1^ on flat surfaces (pods) (Table 1, Supplementary Material Fig. S1). Trace metal analysis of experiment treatment water (filtered leachate) sampled at 28 d showed significantly (*P* < 0.05) higher concentrations of arsenic, cobalt and nickel in certain coal treatments in comparison with control water (Table 2). However, the highest metal concentrations were not always measured in the highest coal treatments. The magnitude of change in dissolved metal concentrations in relation to control seawater was minimal: arsenic varied by 0.3 μg l^−1^; cadmium 0.1 μg l^−1^; cobalt 0.2 μg l^−1^; copper 0.2 μg l^−1^; lead 0.1 μg l^−1^; manganese 0.3 μg l^−1^; molybdenum 0.8 μg l^−1^; nickel 2.4 μg l^−1^; zinc 0.9 μg l^−1^. These findings suggest that metals were not likely contributing to the observed effects.

Although the tanks were moderately turbulent (water flow of 5–10 cm sec^−1^) due to the presence of pumps, the coal particles attached to many surfaces within the tanks, contributing to lower total suspended coal (TSC) exposures in the latter half of the four week pulse (Supplementary Material Fig. S1). While there is limited evidence documenting the concentrations of suspended coal present in seawater during a spill event, our high coal treatment (275 mg coal l^−1^) was lower than the concentrations applied to temperate species in other experimental studies (500–13,500 mg coal l^−1^)^16^,^17^. Moreover, the results of the present experiment may be considered conservative in relation to the broader effects of coal during a spill event as we only investigated the effects of fine coal particles (<63 μm) which are likely to remain in suspension for long periods^3^,^18^. A large spill scenario at sea would also release larger particles that settle more rapidly^3^,^18^, posing further risks of physical damage, including smothering.

### Responses of tropical marine organisms to coal exposure

#### Corals

**Figure 1:**
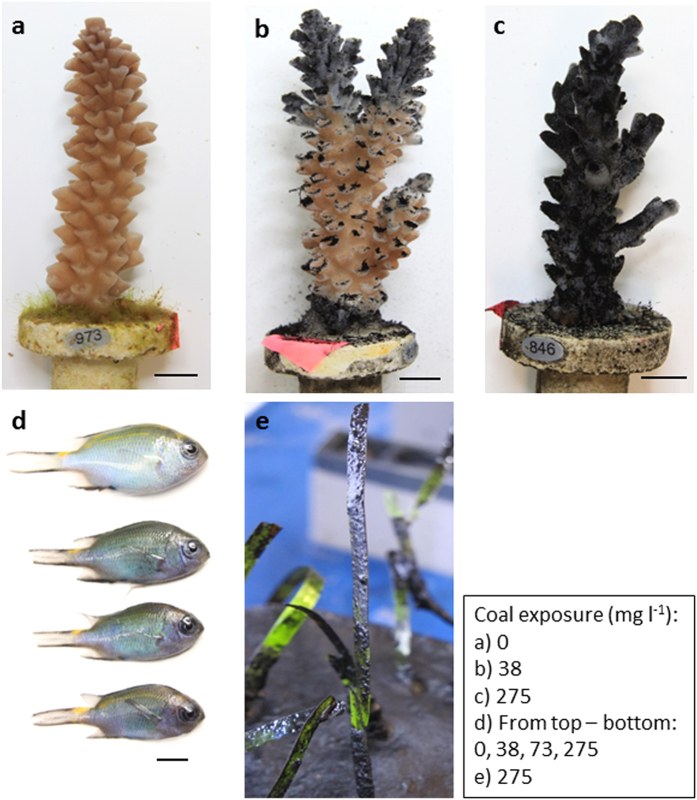
Comparison of three taxa after coal exposure. Stages of coral health degradation after 14 d exposure to 0 mg coal l^−1^ (**a**), 73 mg coal l^−1^ (**b**) and 275 mg coal l^−1^ (**c**). Mucus strands were used to actively remove settled coal (**b**) and coal deposition that exceeded removal efforts resulted in nubbin mortality (**c**). Fish from control vs. coal exposed treatments (0 mg coal l^−1^–275 mg coal l^−1^) after 28 d exposure (**d**). Coal settled onto seagrass leaves and substrate (**e**). Note: No fish were present in the 202 mg coal l^−1^ treatment. All scale bars = 5 mm.

**Figure 2:**
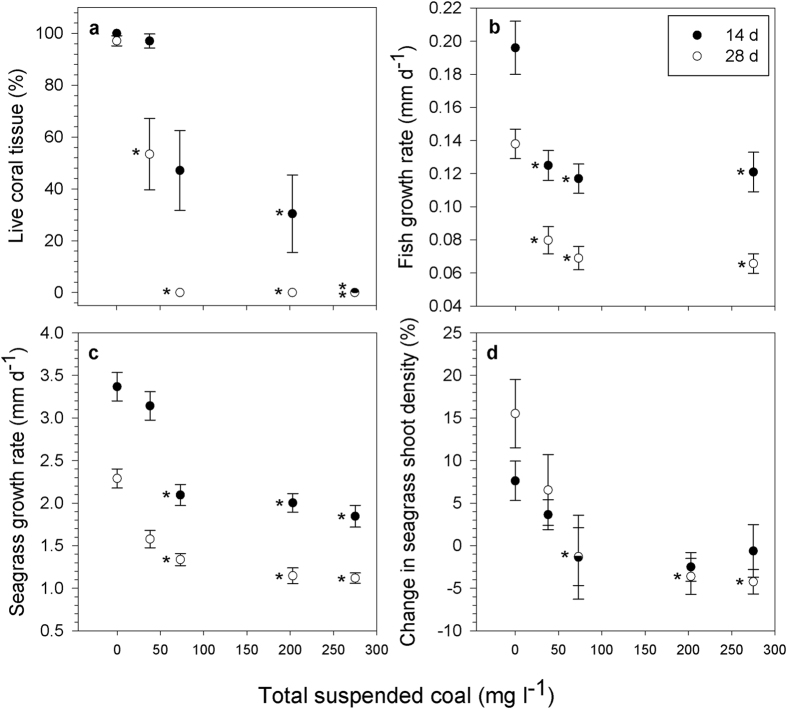
Differences in measures of key demographic rates in relation to coal concentration and exposure duration. Differences in the mean (±s.e.m.) survival of corals (*A. tenuis*) (**a**), growth rates of fish (*A. polyacanthus*) (**b**) and seagrass (*H. uninervis*) (**c**), and percentage change in seagrass shoot density (**d**) at 14 d (*closed circle*) and 28 d (*open circle*) exposure. Asterisks depict a significant difference (*P* < 0.05) between the mean coal treatment and control values. Note: mean change in seagrass shoot density (**d**) is relative to time 0 values at each treatment level in each replicate seagrass pot. Mean values above 0 suggest growth, while values below 0 suggest mortality. No fish were present in the 202 mg coal l^−1^ treatment.

In all coal treatments, particles settled directly onto coral polyps and connecting tissue (i.e., coenosarc, Fig. 1b,c) and the initial response of corals to coal exposure was the release of fine mucus strands, which trapped coal particles and removed them from the tissue surface, similar to the response of corals exposed to sediments^7^,^19^,^20^. Branching corals, such as *A. tenuis,* are often considered among the most resistant morphologies to sedimentation due to their vertical growth^20^, yet despite their active mechanisms for coal removal; some coral tissue died and sloughed off the skeleton within 14 d in all treatments ≥38 mg coal l^−1^ (Figs 1b,c and 2a). The extent of tissue mortality on coral branches differed significantly among coal treatments (Permanova, Pseudo-F_4,10_ = 43.6, *P* = 0.0001), and over time (Permanova, Pseudo-F_1,10_ = 20.5, *P* = 0.0009) (Fig. 2a, see statistical outputs in Supplementary Material Tables S1 and S2). After 14 d of exposure, control and low (38 mg coal l^−1^) coal treatments exhibited significantly lower coral mortality than treatments ≥202 mg coal l^−1^ (Student-t post hoc, Monte Carlo simulation, *P* < 0.05). After 28 d, mortality in all coal treatments ≥38 mg coal l^−1^ was significantly higher than the controls (Student-t post hoc, Monte Carlo simulation, *P* < 0.05). Corals in the control treatment exhibited less than 3% mortality, while 100% tissue mortality occurred in all branches in the three highest coal exposures (Figs 1c and 2a). Corals in the 38 mg coal l^−1^ treatment exhibited significantly lower mortality than corals in treatments ≥73 mg coal l^−1^ (Student-t post hoc, Monte Carlo simulation, *P* < 0.05). Pair-wise comparisons between treatments revealed lowest observed effect concentrations (LOEC) of 202 mg coal l^−1^ and 38 mg coal l^−1^ at 14 and 28 d, respectively. Fitting four-parameter sigmoidal curves to the data revealed lethal concentrations (LC_10_ and LC_50_) of 29 mg coal l^−1^ and 87 mg coal l^−1^ at 14 d, respectively, and 34 mg coal l^−1^ and 36 mg coal l^−1^ at 28 d, respectively (Supplementary Material Fig. S2a).

Coral mortality in response to the suspension of fine coal particles may have a number of causes. The accumulation of coal particles on the vertical tissue could have caused anoxia at the coral-coal interface^21^. Similar surface accumulation of particles was not observed after a month in comparable exposures of *Acropora millepora* branches to fine carbonate sediments^22^ and could indicate either greater adhesion by coal or reduced fitness in corals exposed to coal rather than sediments. The energetic costs of removing deposited particles (including mucus production) may be further exacerbated in the presence of coal by the strong attenuation of light over 14 and 28 d, which would reduce primary production rates by the symbiotic dinoflagellates. Although the corals were fed once per week with *Artemia* nauplii, heterotrophic feeding behaviour may have been altered in smothered sections of coral colonies^19^.

#### Fish

The health of coal-exposed fish was compromised in all coal treatments and differences in fish size and colour were observed over the course of the experiment (Fig. 1d). Fish growth rates varied significantly between coal treatments and controls (Permanova, Pseudo-F_3,8.1_ = 21.7, *P* = 0.01), and over time (Permanova, Pseudo-F_1,8.5_ = 141.4, *P* = 0.0002) (Fig. 2b, see statistical outputs in Supplementary Material Tables S1 and S2). Significant differences in growth occurred within the first 14 d of the experiment, with fish exposed to coal levels ≥38 mg coal l^−1^ showing significantly lower growth rates than control fish, irrespective of coal treatment levels (Student-t post hoc, Monte Carlo simulation, *P* < 0.05, Fig. 2b). Growth inhibition, relative to control fish, at 14 d ranged from 36.1 ± 4.6%–40.2 ± 4.5% and 42.2 ± 6.0%–52.3 ± 4.3% at 28 d (Supplementary Material Fig. S2b). The LOEC on fish growth rates was 38 mg coal l^−1^ at both time points and linear interpolation of the growth data revealed inhibition concentration (IC_10_) estimates of 11 and 9 mg coal l^−1^ at 14 and 28 d, respectively, and IC_50_ estimates of 73 mg coal l^−1^ at 28 d. IC_50_ estimates were not possible at 14 d because growth inhibition was below 50%.

The negative impact of coal on fish growth is consistent with the response of marine fish to increased suspended sediments which is thought to be caused by visual impairment leading to reduced prey capture success and increased foraging time and energy expenditure^23^,^24^. A preliminary post mortem investigation on the coal-exposed fish in this experiment revealed coal in the alimentary tracts, which was mistakenly ingested and could have physically blocked normal feeding and digestion contributing to starvation and debilitation. In addition, it is possible that suspended coal affected fish respiration^25^,^26^, an effect that may have been consistent across all coal treatments.

Despite the considerable effects on fish growth, all coal-exposed fish survived except for two individuals that were exposed to the highest coal treatment of 275 mg coal l^−1^. The lethal effects of suspended sediments on fish are dependent on the particle size, angularity, exposure duration, and are typically observed when concentrations reach ≥hundreds of mg l^−1^ 23,27,28. The survival of fish in the current study, along with the very high LC_50_ (7000 mg coal l^−1^) reported for 8 d coal exposures of juvenile coho salmon^17^, support the notion that coal spills are not likely to cause direct mortality in fish under most coal spill scenarios. However, suspended sediment can prolong reef fish larvae development^29^, negatively influence gill morphology and increase pathogenic bacterial communities on larval gills^30^, suggesting further studies are required to investigate the vulnerability of early life stages of fish to suspended coal. Although mortality was low, certain post-settlement processes are size dependent for reef fishes^31^, suggesting that lower growth rates *in situ* may have later implications on individual survivorship^23^. Moreover, as fecundity of fish is size dependent^31^,^32^, suppressed growth can lower lifetime reproductive output.

#### Seagrass

Coal particles were observed to attach to the seagrass leaves less than 24 h after exposure commenced, and many leaves were completely coated in a film of coal throughout the experiment (Fig. 1e). Coal also accumulated onto the sediment surface in seagrass pots where new shoots develop (Fig. 1e). Significant differences were measured for leaf elongation (Permanova, Pseudo-F_4,10_ = 35.9, *P* = 0.0002) and shoot density (Permanova, Pseudo-F_4,10_ = 9.8, *P* = 0.0002) between experimental treatments. Leaf elongation rates differed significantly over time (Permanova, Pseudo-F_1,10_ = 67.5, *P* = 0.0001) (see statistical outputs in Supplementary Material Tables S1 and S2). Leaf elongation was more sensitive than shoot density and was significantly affected (Student-t post hoc, Monte Carlo simulation, *P* < 0.05) in treatments ≥73 mg coal l^−1^ (LOEC) at both 14 d and 28 d (Fig. 2c). The magnitude of the effect of coal exposure on leaf elongation rates was large, with overall growth inhibited by 6.7 ± 5.0%–45.2 ± 3.8% and 31.1 ± 4.5%–49.5 ± 3.1% relative to controls at 14 and 28 d, respectively (Supplementary Material Fig. S2c). The estimated threshold for impact for this parameter (IC_10_) was 42 mg coal l^−1^ at 14 d and 12 mg coal l^−1^ at 28 d, while the IC_50_ was 275 mg coal l^−1^ at 28 d. IC_50_ estimates were not possible at 14 d because growth inhibition was below 50%. Shoot density continued to increase in control and 38 mg coal l^−1^ treatments throughout the experiment duration, however, was significantly reduced (Student-t post hoc, Monte Carlo simulation, *P* < 0.05) at 28 d in coal treatments ≥73 mg coal l^−1^ (28 d LOEC), with a mean net loss of 1.3 ± 3.4%–4.6 ± 1.4% of shoots at this time point (Fig. 2d).

The coal exposures may have impacted the seagrass in multiple ways. Seagrass requires light to conduct photosynthesis and the light environment was greatly affected by attenuation through the water column (Table 1). Irradiance intensity is a principal factor regulating seagrass growth and shading of surface irradiance to low levels (0.2–4.4 mol m^−2 ^d^−1^) has contributed to reduced leaf elongation and shoot loss in the same species^33^. In addition, the direct coating of leaves with a layer of coal particles is likely to further reduce light penetration^34^. Both types of shading and reduced transport of CO_2_ into the leaves through the coal barrier will limit photosynthetic carbon fixation, chlorophyll *a* production^34^ and inhibit growth^35^. Seagrasses maintain a store of carbohydrates within the root-rhizome complex and this is likely to have enabled slight positive leaf extension over the experimental exposures^36^. Although not directly measured in this experiment, coal exposure can also cause abrasive damage to aquatic plants^16^,^37^.

## Conclusions

While there were differences in sensitivity between the taxa tested here, both sessile and mobile organisms were affected by similar concentrations of coal particles. In most cases the impacts increased with suspended coal concentration and exposure duration. Although this study did not specifically investigate the stress-response pathways, it was clear that coal particles affect corals, fish and seagrass in ways that are similar to the effects of other suspended solids, including: light limitation, direct smothering and reduced feeding efficiency. Despite these similarities, coal particles appear to have more severe effects on corals than other suspended solids. For instance, chronic exposure of corals to fine carbonate sediment in a similar experimental setup resulted in only 11% mortality in branching coral *Acropora millepora* after 84 d exposure to 100 mg l^−1^ (83 mg cm^−2 ^d^−1^ deposition)^22^, while only high levels of acute bottom sand deposition (200 mg cm^−2 ^d^−1^) caused mortality in branching coral *Acropora palmata*^38^. These differences may be due to coal particles attenuating light more strongly and adhering to coral to a greater extent than inorganic particles. While several metals were elevated in coal treatments in comparison with controls, the magnitude of this increase due to leaching was minimal and only the relatively low toxicity element cobalt was detected at concentrations greater than ANZECC guidelines^39^. Our results are consistent with previous studies that showed coal generally does not leach toxic levels of trace metals^10^,^11^. Though not measured in the present study, leaching of PAHs from coal is also generally considered low and to have very low bioavailability^3^,^9^,^40^, suggesting that the measured adverse effects in the present study were primarily due to physical mechanisms.

As global demand for, and marine transport of, coal continues to increase, specific information is needed to effectively manage risks to areas of high conservation value, such as coral reefs and seagrass meadows, that may be impacted by unburnt coal from terrestrial sources and accidental spills. This first study to examine the effects of fine coal particles on tropical marine organisms demonstrates that moderate to high levels of coal contamination can substantially decrease growth and increase mortality of important reef-building coral species, reef fish and seagrass. Further research is warranted to measure the effects of coal contamination on reproduction and early life histories of corals, fish, invertebrates, as well as the effects of ingestion and smothering on sessile benthic organisms. Considering that hydrocarbon markers for coal have been identified up to 180 m offshore in the World Heritage listed GBR^41^, understanding the risks posed by unburnt coal also requires an improved understanding of *in situ* chronic exposures from coastal operations^9^ and potential transport into reef and seagrass systems by wind and currents. The experimental scenario applied in the present study is particularly relevant to shipping accidents, where high concentrations of unburnt coal can be present in water adjacent to globally threatened habitats. The effect thresholds of coal to coral, fish and seagrass, such as those identified here, are critically important for marine park managers, regulators, industry and shipping operators as a basis to improve risk assessments and policy development associated with safer and more sustainable shipment of coal.

## Materials and Methods

### Conceptual basis for experimental design

This experiment was conducted at the indoor facilities of the National Sea Simulator at the Australian Institute of Marine Science (AIMS) in June–July 2014. Thermal coal (sourced from central Queensland, Australia) was crushed, milled and sieved to isolate particles <63 μm. Organisms were exposed to five coal treatment levels (0–275 mg coal l^−1^) in custom 55 l flow-through tanks (n = 3 per treatment) that were designed to maintain particles in suspension, with twice daily turnovers of water. Coal suspension was aided by the sloped tank base that used gravity to draw settled particles towards an external pump that re-circulated/suspended particles, and an air stone within the tank that aided particle movement (see Supplementary Material, Fig. S3). Each treatment tank contained 9 coral (*A. tenuis*) fragments (5 cm length), 10 fish (*A. polyacanthus*, ~11 weeks old) and 3 pots of seagrass (*H. uninervis*, average 33 shoots per pot growing in sediment), with the exception of the 202 mg coal l^−1^ treatments which contained no fish.

### Response variables monitored for experimental organisms during coal exposure

Growth and mortality were assessed for the study species in each experimental treatment and at two time points during the experiment (14 and 28 d). For seagrass, leaf elongation was used as a proxy for growth. Between each sampling point 5 leaves per pot (n = 45 per treatment) were haphazardly chosen and pierced 2 times with an insulin needle at the top of the sheath. The distance between the sheath holes and needle scars in the leaf were measured using callipers. New holes were made approximately 1 week prior to each sampling period. Measurements were converted into growth rate per day (mm d^−1^). For fish, the standard length was measured in each fish 5 d prior to the commencement of the experiment and again after 14 and 28 d of coal exposure. Each fish was tagged with an individual fluorescent marker by subcutaneous injection of an elastomer dye with an insulin needle^23^ and fish were measured in seawater-filled zip lock bags with hand held calipers. Seagrass and fish growth inhibition values were calculated relative to the mean control growth rates at 14 and 28 d, respectively.

Coral mortality was measured at each sampling interval. Nine random coral fragments were sacrificed by snap freezing with liquid nitrogen then photographed at 2 different (non-overlapping) angles next to a scale bar. To avoid confounding irregularities at the bases of fragments due to fragmentation and gluing, the bottom 3 mm of each branch was omitted from the measurement. ImageJ software (U.S. NIH, MD, USA http://rsb.info.nih.gov/ij/) was used to analyse the proportion of dead tissue on each coral fragment. Tissue was categorized as 1) alive = presence of pigmented or bleached tissue; 2) dead = sloughed tissue (visible skeletal structure) or coal smothered skeleton. For the seagrass, loss of above ground shoot density was used as a proxy for mortality. Prior to the commencement of the experiment and at each sampling interval individual seagrass shoots were counted in each pot. Change in shoot density was calculated by subtracting the shoot count of each pot at a time point from the initial (time 0) shoot count of the same pot. These values were converted into percentage change in shoot density relative to time 0 for each respective treatment level. Finally, fish mortality was assessed at each sampling interval by counting the number of live fish in each tank. All experimental protocols involving fish were approved by James Cook University and the methods were carried out in accordance with the approved James Cook University animal ethics guidelines.

### Water quality parameters

Water quality parameters were measured in each treatment tank throughout the 28 d exposure period. Total suspended solid (TSS) sampling was performed 6–7 times per fortnight on 500 ml aliquots from each tank (n = 1 per tank) during the experimental period. Water was shaken and filtered through pre-weighed filters (0.7 μm glass microfibre) that were then rinsed with deionized water and oven dried (60 °C) until a constant weight was maintained. The gain in weight of each filter was multiplied by 2 to express the TSS in mg l^−1^. Since other organic materials, such as algae, faecal matter and uneaten fish food, were present in all of the experimental tanks including the control treatment, the mean TSS measured in control tanks was subtracted from coal treatments to derive measurements of total suspended coal (TSC; Supplementary Fig. S1a). Temperature was measured 5 times per fortnight (n = 1 per tank) with a thermometer and light attenuation, expressed as photosynthetically active radiation (PAR) was measured weekly (n = 1 per tank) with a Li-250A light meter (Li-cor, Lincoln NE, USA) at the height of the corals and seagrass in the experimental tanks (approximately 25 cm below the water surface). Dissolved oxygen saturation was measured at the start of the experiment followed by twice per week (n = 1 per tank) using a Hach Probe (HQ 40 d) and pH was measured on 3 occasions (n = 1 per tank) using a potentiometric pH probe (console: OAKTON, USA; pH probe: EUTECH, USA).

Coal deposition rates were measured in each tank using 2 methods (Supplementary Fig. S1b,c). The first involved small sediment traps (n = 3 per tank) (20 ml glass vials, 15 mm opening diameter, 58 mm height) with the top at a height similar to the corals^22^. The second method used flat-surfaced sediment pods (n = 1 per tank), which allow for re-suspension of particles^42^. Both traps (sampled weekly) and pods (sampled 4–5 times per fortnight) were collected 24 h after deployment and contents were filtered through pre-weighed filters (0.7 μm glass microfibre) that were rinsed with deionized water and oven dried (60 °C) until a constant weight was maintained for determination of deposition rate. Similar to TSC measurements, the mean weight of organic material deposited onto control filters was subtracted from the mean coal deposition values in each treatment in order to present a measurement of coal deposition only.

Water samples were taken at 28 d to assess the potential contamination by trace metals (Co, As, Cd, Cu, Pb, Mn, Mo, Ni, Zn). Metal analysis was conducted at Charles Darwin University (Australia) using inductively coupled plasma mass spectrometry (ICP-MS). Water samples (0.45 μm syringe filtered leachate, 150 ml) were taken from each treatment (n = 3; 3 × 50 ml per tank, which was pooled for each treatment replicate). PAHs were not detected or bioavailable in previous coal seawater leaching studies^3^,^40^ and were not analysed here.

### Statistical analysis

To evaluate organism responses to coal particles a multifactor analysis of variance between 14 and 28 d was implemented based on permutations using the PERMANOVA routine of PRIMER (Version 6.0). Euclidean Distance was used as the similarity measure (with 9999 permutations) and pair-wise comparisons were made with the Student t-test with Monte Carlo simulations used when unique permutations were <1000. Coral mortality (%) data was arcsine square root transformed prior to analysis. The n for each taxa in the PERMANOVA analysis were as follows: coral (n = 9 per treatment per time point), fish (n = 23–29), and seagrass (growth n = 45 leaves per treatment per time point; change in shoot density n = 9 pots per treatment per time point). The factors analysed were: coal concentration (5 fixed), exposure time (2 fixed), tank (3 random: nested within concentration), with the addition of replicates (e.g., replicated seagrass pots, random: nested within tank) where appropriate (Supplementary Material Table S1). Four-parameter sigmoidal curves were fitted to coral mortality data to estimate lethal concentration (LC_10_ and LC_50_) values for mortality using GraphPad Prism (Version 6.0). Concentrations resulting in mean inhibition of growth (IC_10_ and IC_50_) were estimated for fish and seagrass using linear interpolation in SigmaPlot (Version 11.0). Analysis of variance (one-way ANOVA) was used to compare means of trace elements between coal treatments in SigmaPlot (Version 11.0). Elements (Co and Pb) that did not meet the assumptions of normality (based on Shapiro-Wilk normality test) were log-transformed.

## Additional Information

**How to cite this article**: Berry, K. L. E. *et al*. Simulated coal spill causes mortality and growth inhibition in tropical marine organisms. *Sci. Rep.* **6**, 25894; doi: 10.1038/srep25894 (2016).

## Supplementary Material

Supplementary Information

## Figures and Tables

**Table 1:** Summary of water quality parameters.

Treatment	TSC	Light	Light attenuation (%)	Coal deposition (vials)	Coal deposition (pods)	Temperature	Dissolved oxygen	pH
	n = 35–39	n = 12	n = 12	n = 36	n = 22–27	n = 30	n = 27	n = 9
Control	0	177 ± 8.59	–	0	0	26 ± 0.15	8.5 ± 0.02	8.1 ± 0.02
Low	38 ± 6	99 ± 9.39	44	11 ± 1.99	2.3 ± 0.24	26 ± 0.13	8.3 ± 0.03	8.0 ± 0.00
Moderate	73 ± 11	21 ± 3.76	88	38 ± 4.94	11 ± 1.48	26 ± 0.10	8.3 ± 0.02	8.0 ± 0.00
Medium	202 ± 32	1.9 ± 0.70	99	126 ± 24	25 ± 3.16	27 ± 0.09	8.2 ± 0.02	8.0 ± 0.00
High	275 ± 36	1.1 ± 0.32	99	241 ± 37	46 ± 4.25	26 ± 0.10	8.3 ± 0.02	8.0 ± 0.00

Mean (±s.e.m.) total suspended coal (TSC) (mg l^−1^), light (PAR, μmol photons m^−2 ^s^−1^), light attenuation (% rel. to 0 mg coal l^−1^), coal deposition rates (mg cm^−2^ day^−1^) in glass vials and deposition pods, temperature (°C), dissolved oxygen (mg l^−1^) and pH. Note: Mean deposition of particulate matter in control treatments (7.3 mg l^−1^ for TSC, 5.2 and 0.7 mg cm^−2^ day^−1^ for vials and pods, respectively) was subtracted from all coal treatments to depict only coal suspension and deposition. Variation in TSC and deposition over time are presented in Supplementary Material Fig. S1. n = total replicates per treatment over the experiment duration.

**Table 2:** Elemental analysis (μg l^−1^) from water samples (n = 3) in each treatment (mean ± s.e.m.).

Element	Leach test (μg l^−1^) from each coal treatment				
	Control (0 mg l^−1^)	Low (38 mg l^−1^)	Moderate (73 mg l^−1^)	Medium (202 mg l^−1^)	High (275 mg l^−1^)
Arsenic (As)	1.2 ± 0.1	1.4 ± 0.1	1.5 ± 0.1*	1.4 ± 0.1	1.4 ± 0.0
Cadmium (Cd)	0.1 ± 0.0	0.1 ± 0.0	0.1 ± 0.0	0.0 ± 0.0	0.1 ± 0.0
Cobalt (Co)	0.0 ± 0.0	0.1 ± 0.0*	0.1 ± 0.0*	0.2 ± 0.0*	0.1 ± 0.0*
Copper (Cu)	0.3 ± 0.1	0.5 ± 0.1	0.5 ± 0.1	0.5 ± 0.1	0.5 ± 0.3
Lead (Pb)	0.0 ± 0.0	0.1 ± 0.0	0.1 ± 0.0	0.0 ± 0.0	0.0 ± 0.0
Manganese (Mn)	0.3 ± 0.1	0.3 ± 0.1	0.4 ± 0.1	0.6 ± 0.1	0.6 ± 0.2
Molybdenum (Mo)	11.3 ± 0.2	11.0 ± 0.5	11.4 ± 0.5	12.0 ± 0.2	12.1 ± 0.1
Nickel (Ni)	0.5 ± 0.1	1.1 ± 0.2	1.8 ± 0.4	2.9 ± 0.8*	2.6 ± 0.5
Zinc (Zn)	2.1±0.7	2.9 ± 1.1	3.0 ± 0.9	2.5 ± 0.3	2.9 ± 0.8

Coal treatments where levels were significantly different from the control treatment (ANOVA, one-way analysis of variance) are depicted with a*.

## References

[b1] International Energy Agency (IEA). Coal information (2014 Edition). *Technical Report*, 748 pg. Available for purchase at: http://www.iea.org/publications/.

[b2] Hughes T. P., Day J. C. & Brodie J. Securing the future of the Great Barrier Reef. Nat. Clim. Change 5, 508–511 (2015).

[b3] Jaffrennou C. . Simulations of accidental coal immersion. Mar. Pollut. Bull 54, 1932–1939 (2007). 17964611 10.1016/j.marpolbul.2007.08.017

[b4] Department of Environmental Affairs Republic of South Africa. *Department of environmental affairs grants permit for coal from MV Smart to be dumped into the ocean. Media release.* (2013). Available at: https://www.environment.gov.za/mediarelease/coalfrom_mvsmart_dumped. (Accessed: 11 September 2014).

[b5] Australian Transport Safety Bureau (ATSB). Independent investigation into the grounding of the Chinese registered bulk carrier Shen Neng 1 on Douglas Shoal, Queensland 3 April 2010. Transport Safety Report No. 274 MO -2010-003, 70 (2010).

[b6] Hyslop B. T., Davies M. S., Arthur W., Gazey N. J. & Holroyd S. Effects of colliery waste on littoral communities in north-east England. Environ. Pollut. 96, 383–400 (1997). 15093404 10.1016/s0269-7491(97)00039-0

[b7] Rogers C. S. Responses of coral reefs and reef organisms to sedimentation. Mar. Ecol. Prog. Ser. 62, 185–202 (1990).

[b8] Fabricius K. E. Effects of terrestrial runoff on the ecology of corals and coral reefs: review and synthesis. Mar. Pollut. Bull 50, 125–146 (2005). 15737355 10.1016/j.marpolbul.2004.11.028

[b9] Ahrens M. J. & Morrisey D. J. Biological effects of unburnt coal on the marine environment. Oceanog. Mar. Biol. 43, 69–122 (2005).

[b10] Cabon J. Y., Burel L., Jaffrennou C., Giamarchi P. & Bautin F. Study of trace metal leaching from coals into seawater. Chemosphere 69, 1100–1110 (2007). 17521696 10.1016/j.chemosphere.2007.04.018

[b11] Lucas S. A. & Planner J. Grounded or submerged bulk carrier: the potential for leaching of coal trace elements to seawater. Mar. Pollut. Bull 64, 1012–1017 (2012). 22417390 10.1016/j.marpolbul.2012.02.001

[b12] Bryan G. W. The effects of heavy metals (other than mercury) on marine and estuarine organisms. P. Roy. Soc. Lond. B Biol. 177, 389–410 (1971). 10.1098/rspb.1971.0037 4396393

[b13] Di Toro D. M. & McGrath J. A. Technical basis for narcotic chemicals and polycyclic aromatic hydrocarbon criteria. II. Mixtures and sediments. Environ. Toxicol. Chem. 19, 1971–1982 (2000).

[b14] Eisler R. Polycyclic aromatic hydrocarbon hazards to fish, wildlife and invertebrates: a synoptic review. US fish and wildlife service biological report 85.1.11, 81 (1987).

[b15] Kennish M. J. Pollution Impacts on Marine Biotic Communities. (ed. Kennish M. J.) 310 (CRC Press, 1998).

[b16] Lewis K. The effect of suspended coal particles on the life forms of the aquatic moss *Eurhynchium riparioides* (Hedw.). Freshwater Biol. 3, 251–257 (1973).

[b17] Pearce B. C. & McBride J. A preliminary study on the occurrence of coal dust in Roberts Bank sediments and the effects of coal dust on selected fauna. Technical Report Series No. PAC/T-77-17, Fisheries and Environment Canada, Water Quality Division 50 (1977).

[b18] Johnson R. & Bustin R. M. Coal dust dispersal around a marine coal terminal (1977–1999), British Columbia: The fate of coal dust in the marine environment. Intl. J. Coal Geol. 68, 57–69 (2006).

[b19] Peters E. C. & Pilson M. E. Q. A comparative study of the effects of sedimentation on symbiotic and asymbiotic colonies of the coral *Astrangiadanae* Milne Edwards and Haime 1849. J. Exp. Mar. Biol. Ecol. 92, 215–230 (1985).

[b20] Stafford-Smith M. G. & Ormond R. F. G. Sediment-rejection mechanisms of 42 species of Australian scleractinian corals. Mar. Freshwater Res. 43, 683–705 (1992).

[b21] Weber M. . Mechanisms of damage to corals exposed to sedimentation. P. Natl. Acad. Sci. USA 109, E1558–1567 (2012). 10.1073/pnas.1100715109 PMC3386076 22615403

[b22] Flores F. . Chronic exposure of corals to fine sediments: lethal and sub-lethal impacts. PloS One 7, e37795–37800 (2012). 22662225 10.1371/journal.pone.0037795 PMC3360596

[b23] Wenger A. S., Johansen J. L. & Jones G. P. Increasing suspended sediment reduces foraging, growth and condition of a planktivorous damselfish. J. Exp. Mar. Biol. Ecol. 428, 43–48 (2012).

[b24] Sigler J. W., Bjornn T. C. & Everest F. H. Effects of chronic turbidity on density and growth of steelheads and coho salmon. T. Am. Fish Soc. 113, 142–150 (1984).

[b25] Hughes G. M. Coughing in the rainbow trout (*Salmo gairdneri*) and the influence of pollutants. Rev. Suisse Zool 82, 47–64 (1975). 1182042 10.5962/bhl.part.78257

[b26] Sutherland A. & Meyer J. Effects of increased suspended sediment on growth rate and gill condition of two southern Appalachian minnows. Environ. Biol. Fish. 80, 389–403 (2007).

[b27] Birtwell I. K. C. The effects of sediment on fish and their habitat. Canadian Stock Assessment Secretariat Research Document. Fisheries and Oceans Canada. Pacific Science Advice and Review Committee Habitat Subcommittee 99–139 (1999).

[b28] Lake R. G. & Hinch S. G. Acute effects of suspended sediment angularity on juvenile coho salmon (Oncorhynchus kisutch). Can. J. Fish Aquat. Sci. 56, 862–867 (1999).

[b29] Wenger A. S. . Suspended sediment prolongs larval development in a coral reef fish. J. Exp. Biol. 217, 1122–1128 (2014). 24311818 10.1242/jeb.094409

[b30] Hess S., Wenger A. S., Ainsworth T. D. & Rummer J. L. Exposure of clownfish larvae to suspended sediment levels found on the Great Barrier Reef: Impacts on gill structure and microbiome. Scientific Reports 5, 10561, doi: 10.1038/srep10561 (2015). 26094624 PMC5392994

[b31] Perez K. O. & Munch S. B. Extreme selection on size in the early lives of fish. Evolution 64, 2450–2457 (2010). 20298462 10.1111/j.1558-5646.2010.00994.x

[b32] Hislop J. R. G. The influence of maternal length and age on the size and weight of the eggs and the relative fecundity of the haddock, *Melanogrammus aeglefinus*, in British waters. J. Fish Biol. 32, 923–930 (1988).

[b33] Collier C. J., Waycott M. & Ospina A. G. Responses of four Indo-West Pacific seagrass species to shading. Mar. Pollut. Bull 65, 342–354 (2012). 21741666 10.1016/j.marpolbul.2011.06.017

[b34] Naidoo G. & Chirkoot D. The effects of coal dust on photosynthetic performance of the mangrove, Avicennia marina in Richards Bay, South Africa. Environ. Pollut. 127, 359–366 (2004). 14638296 10.1016/j.envpol.2003.08.018

[b35] Ralph P. J., Durako M. J., Enríquez S., Collier C. J. & Doblin M. A. Impact of light limitation on seagrasses. J. Exp. Mar. Biol. Ecol. 350, 176–193 (2007).

[b36] Alcoverro T., Manzanera M. & Romero J. Annual metabolic carbon balance of the seagrass *Posidonia oceanica*: the importance of carbohydrate reserves. Mar. Ecol. Prog. Ser. 211, 105–116 (2001).

[b37] Hyslop B. T. & Davies M. S. Evidence for abrasion and enhanced growth of *Ulva lactuca L*. in the presence of colliery waste particles. Environ. Pollut. 101, 117–121 (1998). 15093104 10.1016/s0269-7491(98)00006-2

[b38] Rogers C. S. Sublethal and lethal effects of sediments applied to common Caribbean reef corals in the field. Mar. Pollut. Bull 14, 378–382 (1983).

[b39] Australian and New Zealand Environment and Conservation Council (ANZECC). Australian and New Zealand Guidelines for Fresh and Marine Water Quality 1, 314 (2000).

[b40] Bender M. E., Roberts M. H. Jr. & deFur P. O. Unavailability of polynuclear aromatic hydrocarbons from coal particles to the eastern oyster. Environ. Pollut. 44, 243–260 (1987). 15092764 10.1016/0269-7491(87)90202-8

[b41] Burns K. & Brinkman D. Organic biomarkers to describe the major carbon inputs and cycling of organic matter in the central Great Barrier Reef region. *Estuar*. Coast Shelf S. 93, 132–141 (2011).

[b42] Field M. E., Chezar H. & Storlazzi C. D. SedPods: a low-cost coral proxy for measuring net sedimentation. Coral Reefs 32, 155–159 (2013).

