# Supplementary material for: Simulated coal spill causes mortality and growth inhibition in tropical marine organisms
Source: Sci Rep. 2016 May 13;6:25894. doi: 10.1038/srep25894 (PMC4865754; doi:10.1038/srep25894)
Supplement: Supplementary Information [file srep25894-s1.pdf]

Simulated coal spill causes mortality and growth inhibition in tropical marine organisms

Kathryn L. E. Berry[1,2,3,4*], Mia O. Hoogenboom[1,4], Florita Flores[2], Andrew P. Negri[2]

[1]College of Marine and Environmental Sciences, James Cook University, Townsville, Queensland, Australia.
[2]Australian Institute of Marine Science, Townsville, Queensland, Australia.
[3]AIMS@JCU, Australian Institute of Marine Science and James Cook University, Townsville, Queensland, Australia.
[4]ARC Centre of Excellence in Coral Reef Studies, James Cook University, Townsville, Queensland, Australia.

*kathryn.berry@jcu.edu.au

Supplementary Material

Study Species and Sampling Design

Acropora tenuis is a common Indo-Pacific branching coral species frequently found at both inshore and offshore reefs of the Great Barrier Reef (GBR). Three *A. tenuis* colonies were collected from Davies Reef in the GBR lagoon (-18.789067, 147.733951) at a depth of 4 - 6 m. Coral colonies were maintained in 1000 l indoor flow-through holding tanks (27°C, salinity 35.8 ± 0.03 PSU, 12h light:dark photoperiod at ~200 µmol photons m^{-1} s^{-1}) at the National Sea Simulator (Australian Institute of Marine Science, AIMS, Townsville) prior to the experiment. After 1 week of acclimation to indoor conditions, coral colonies were cut into fragments approximately 5 cm in length. Fragments were glued to calcium carbonate pegs and left for a 6 week recovery/acclimation period.

The seagrass species *Halodule uninervis* is commonly found in coastal Queensland environments, including port areas. *H. uninervis* was collected at Cockle Bay, Magnetic Island (-19.198578, 146.791696). Cores of intact seagrass were placed in plant pots lined with a plastic bag which was filled with seawater and sealed during transportation to AIMS. Seagrasses were re-potted within 24 hours and maintained in 150 l indoor flow-through holding tanks (27°C, salinity 35.8 ± 0.03 PSU, 12h light:dark photoperiod at ~200 µmol photons m^{-1} s^{-1}) at the National Sea Simulator. Seagrasses were acclimated to the laboratory conditions 4 weeks prior to the commencement of the experiment.

Eight-week-old *Acanthochromis polyacanthus* were sourced from a captive breeding program at the Marine and Aquaculture Research Facilities Unit, James Cook University, Townsville. Fish were acclimated for 2 weeks to temperature-controlled (27°C, salinity 35.8 ± 0.03 PSU, 12h light:dark photoperiod at ~200 µmol photons m^{-1} s^{-1}) laboratory conditions. Each fish was tagged with an individual fluorescent marker by subcutaneous injection of an elastomer dye with an insulin needle and were left to recover for one week[1]. Fish were randomly assigned to experimental tanks (n = 10 per tank) and were fed once per day with 4 mg of crushed INVE 5/8 enriched food per fish[1] and also had access to the *Artemia* nauplii provided to the corals. Food was also added to the treatment without fish so that corals were exposed to the same food regime.

Water quality parameters

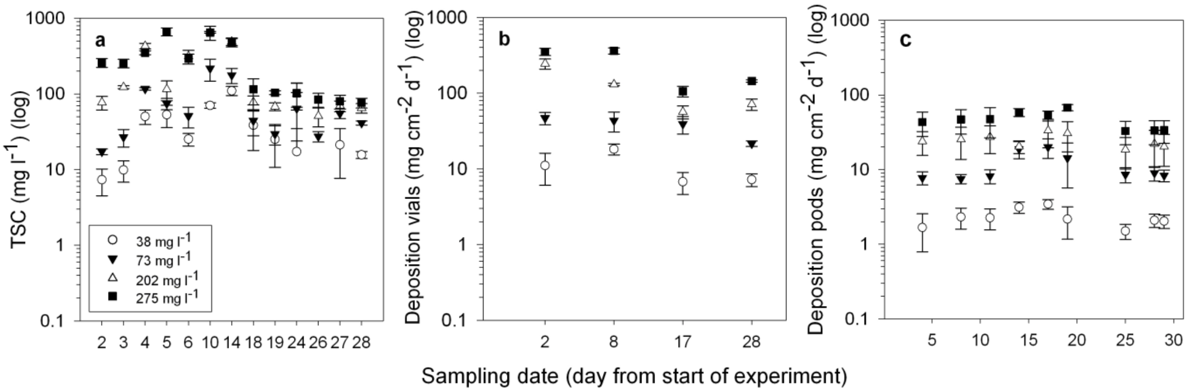

Figure S1, related to Table 1. Suspended and deposited coal. Mean (± s.e.m.) total suspended coal (TSC, a) and coal deposition using sediment vials (b) and pods (c) in all treatments over the experiment duration. Variation of TSC within tanks ranged from 33 - 120% (based on coefficient of variation) and was approximately equivalent to the variation in mean values among tanks within treatments (40 - 99%). TSC values in the coal treatments peaked between 4 - 14 days, after which concentrations stabilised. In general, TSC values gradually declined (by an average of 28 - 78%) between 14 d and 28 d of the experiment due to increased adherence of coal onto sides of aquaria and organisms, as well as flocculation and settlement.

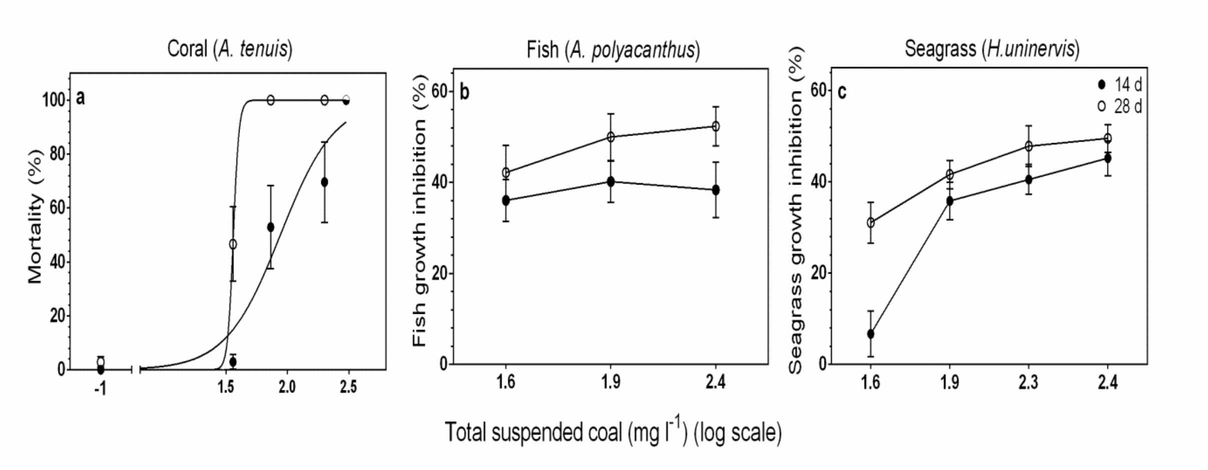

Figure S2. Estimates of lethal and sub-lethal coal concentrations. Mean (± s.e.m.) concentration-response relationship of coral tissue mortality (a) with coal exposure and estimates of growth inhibition in fish (b) and seagrass (c) at 14 d (closed circles) and 28 d (open circles). Lethal concentration (LC_{10} and LC_{50}) values for corals (a) and concentrations resulting in growth inhibition of 10% and 50% of the tested population (IC_{10} and IC_{50}) for fish (b) and seagrass (c) were estimated using linear interpolation. Concentrations are plotted on a log scale.

Experimental system

The flow-through coal delivery system was based on that described in Flores et al.[2] (Fig. S3). The base of each aquarium sloped (36°) towards the front of the tank to reduce particles accumulating on the bottom. An external pump (Eheim Compact+ 3000 at 3000 l h^{-1}: Eheim GmbH, Germany) suctioned particles from the lowest point at the base of each aquarium and resuspended the particles at the back of the tank. Re-suspension and dissolved oxygen saturation, was further maintained with an air stone situated at the rear of each tank. Fresh seawater (filtered to <1 µm) was added at a rate of 4 l h^{-1} to each tank by irrigation dripper (2 water turnovers per tank per day). Experimental coal concentrations were maintained by pulsing coal solutions from highly concentrated stocks (120 - 1200 mg l^{-1}) of suspended coal (in 238 l fibreglass tanks) into each experimental tank (10 pulses of 80 ml stock suspension per h^{-1}, Fig. S3). The coal stock suspension was maintained using an external pump (Eheim Compact 1260 at 2400 l h^{-1}: Eheim GmbH, Germany) that suctioned coal at the stock tank base and delivered it via PVC pipe back to the top of the stock tank, thus maintaining coal particles in suspension (Fig. S3).

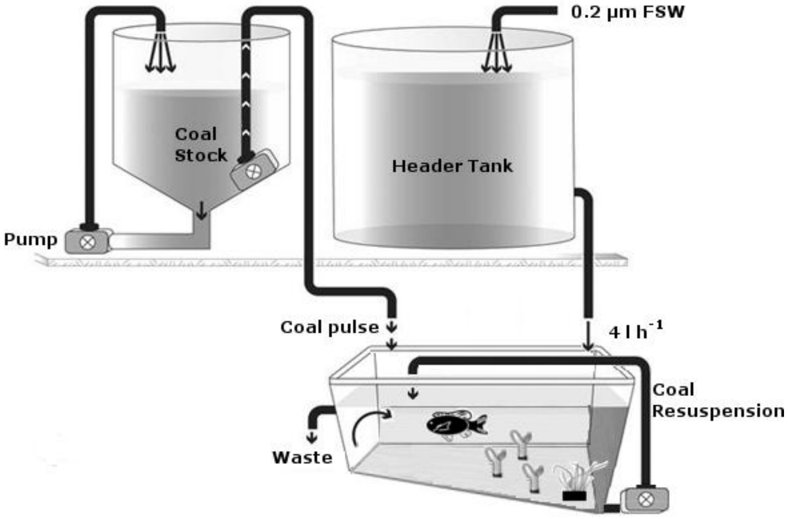

Figure S3. Schematic diagram of experimental system. Adapted from Flores et al.[2]

Table. S1 Statistical outputs of organism response variables. Abbreviations: df = degrees of freedom, SS = sum of squares, MS = mean squares, Co = colony, Fi = fish, Po = pot, Ta = tank, Tr = treatment, Ti = time (14 d and 28d), () = nested factors, x = interaction, P = P value, perm = Permanova, Perms = permutations, CV = coefficient of variation, * = significant p-value.

Variable	Source	df	SS	MS	Pseudo-F	P(perm)	Unique Perms	% CV
Coral mortality	Tr	4	30.3	7.6	43.6	0.0001*	9464	61.8
	Ti	1	3.5	3.5	20.5	0.0009*	9835	11.2
	Ta(Tr)	10	1.7	0.2	1.4	0.212	9949	1.3
	Tr x Ti	4	2.0	0.5	3.0	0.0738	9952	5.7
	Co(Ta(Tr))	30	3.7	0.1	1.4	0.2008	9926	2.4
	Ti x Ta(Tr)	10	1.7	0.2	1.9	0.086	9944	4.1
Fish growth	Tr	3	0.2	0.07	21.7	0.01*	9959	23.4
	Ti	1	0.1	0.1	141.4	0.0002*	9803	24.9
	Ta(Tr)	8	0.02	0.003	1.1	0.3521	9934	0.3
	Tr x Ti	3	0.002	0.0005	0.5	0.6688	9959	0
	Ti x Ta(Tr)	8	0.007	0.0009	0.3	0.9446	9945	0
Seagrass growth	Tr	4	116.1	29.0	35.9	0.0002*	9925	19.6
	Ti	1	110.8	110.8	67.5	0.0001*	9830	30.4
	Ta(Tr)	10	8.1	0.8	0.6	0.7978	9940	0
	Po x Ti	4	11.0	2.7	1.7	0.2363	9964	1.5
	Po(Ta(Tr))	30	39.8	1.3	2.5	0.0003*	9914	5.0
	Ti x Ta(Tr)	10	16.4	1.6	1.4	0.2479	9939	1.8
	Ti x Po(Ta(Tr))	30	36.1	1.2	2.3	0.0003*	9896	8.4
Seagrass shoot density	Tr	4	341.4	85.4	9.8	0.0002*	2215	28.9
	Ti	1	4.4	4.4	0.9	0.3755	9691	0
	Ta(Tr)	10	87.2	8.7	0.6	0.7759	9926	0
	Tr x Ti	4	44.8	11.2	2.2	0.1447	9952	4.6
	Po(Ta(Tr))	30	413.3	13. 8	2.4	0.0097*	9909	27.1
	Ta(Tr) x Ti	10	50.8	5.1	0.9	0.5572	9938	0

Table S2. Statistical outputs of pair-wise comparisons (Student-t post hoc analysis) between treatment levels. Abbreviations: A = 0 mg coal l^{-1}, B = 38 mg coal l^{-1}, C = 73 mg coal l^{-1}, D = 202 mg coal l^{-1}, E = 275 mg coal l^{-1}, d = day, P = P value, perm = Permanova, Perms = permutations, MC = Monte Carlo, Denom 0 = denominator equals 0, * = significant p-value.

	14 d					28 d				
**Variable**	**Treatments**	**t**	**P(perm)**	**Perms**	**P(MC)**	**Treatments**	**t**	**P(perm)**	**Perms**	**P(MC)**
Coral mortality	A, B	1.4	0.4031	2	0.2285	A, B	9.0	0.1009	10	0.001*
	A, C	2.5	0.0978	4	0.0655	A, C	20.7	0.1004	4	0.0001*
	A, D	4.6	0.0972	4	0.0114*	A, D	20.7	0.1001	4	0.0001*
	A, E	Denom 0				A, E	20.7	0.1021	4	0.0001*
	B, C	2.3	0.1009	10	0.0824	B, C	19.8	0.1035	4	0.0001*
	B, D	4.2	0.0988	10	0.0133*	B, D	19.8	0.0989	4	0.0001*
	B, E	29.0	0.1010	4	0.0001*	B, E	19.8	0.0961	4	0.0001*
	C, D	0.5	0.8032	10	0.6561	C, D	Denom 0			
	C, E	1.9	0.3998	2	0.1268	C, E	Denom 0			
	D, E	1.9	0.0934	4	0.1309	D, E	Denom 0			
Fish growth	A, B	4.6	0.0447	358	0.0099*	A, B	5.3	0.0183	716	0.0057*
	A, C	5.6	0.0338	360	0.0044*	A, C	11.9	0.1009	719	0.0001*
	A, E	4.0	0.0892	692	0.0138*	A, E	12.0	0.0959	719	0.0004*
	B, C	0.8	0.4634	90	0.4488	B, C	0.9	0.4848	720	0.4382
	B, E	0.3	0.7736	357	0.7690	B, E	1.3	0.1547	719	0.2673
	C, E	0.2	0.8788	356	0.8546	C, E	0.7	0.4967	719	0.5223
Seagrass growth	A, B	0.8	0.5052	10	0.4541	A, B	2.8	0.1011	10	0.0549
	A, C	4.1	0.0992	10	0.0151*	A, C	8.2	0.1034	10	0.001*
	A, D	6.1	0.0997	10	0.0032*	A, D	5.6	0.0986	10	0.0059*
	A, E	5.8	0.1007	10	0.0044*	A, E	17.0	0.1034	10	0.0002*
	B, C	3.5	0.1010	10	0.0257*	B, C	0.9	0.6009	10	0.4261
	B, D	5.4	0.0989	9	0.0053*	B, D	1.2	0.2968	10	0.2856
	B, E	5.1	0.1038	10	0.0066*	B, E	1.7	0.0991	10	0.1680
	C, D	0.7	0.7016	10	0.5352	C, D	0.7	0.5998	10	0.5530
	C, E	1.2	0.3895	10	0.3138	C, E	1.6	0.2991	10	0.1809
	D, E	0.8	0.6999	10	0.4739	D, E	0.2	0.8973	10	0.8574
Seagrass shoot density	A, B	0.9	0.5022	10	0.4317	A, B	2.1	0.0940	10	0.1097
	A, C	2.3	0.1986	6	0.0824	A, C	3.2	0.1042	9	0.0289*
	A, D	2.6	0.0992	9	0.0544	A, D	4.3	0.0990	10	0.0131*
	A, E	1.9	0.1961	8	0.1323	A, E	4.2	0.1049	7	0.0133*
	B, C	1.8	0.1880	8	0.1466	B, C	2.1	0.1953	8	0.1016
	B, D	2.3	0.1959	5	0.0786	B, D	5.5	0.1042	10	0.0052*
	B, E	1.3	0.2947	5	0.2529	B, E	5.9	0.1036	7	0.0062*
	C, D	0.1	1.0000	5	0.9109	C, D	0.7	0.6989	8	0.5047
	C, E	0.5	0.6977	7	0.6742	C, E	0.6	0.7021	7	0.5711
	D, E	0.5	0.8041	7	0.6661	D, E	0.5	1.0000	2	0.6381

Supplemental References

1 Wenger, A. S., Johansen, J. L. & Jones, G. P. Increasing suspended sediment reduces foraging, growth and condition of a planktivorous damselfish. *J. Exp. Mar. Biol. Ecol.* **428**, 43-48 (2012).

2 Flores, F. *et al.* Chronic exposure of corals to fine sediments: lethal and sub-lethal impacts. *PloS One* **7**, e37795-37800 (2012).
